# RIDA®GENE *Helicobacter pylori* PCR on the ELITe InGenius System

**DOI:** 10.1007/s10096-023-04563-3

**Published:** 2023-03-16

**Authors:** Lucie Bénéjat, Astrid Ducournau, Chloé Domingues Martins, Emilie Bessède, Philippe Lehours

**Affiliations:** 1 French National Reference Center for Campylobacters & Helicobacters, Bordeaux Hospital University Center, 33300 Bordeaux, France; 2 Univ. Bordeaux, INSERM, UMR1312 Bordeaux Institute of Oncology, BRIC, 33076 Bordeaux, France; 3 CHU Pellegrin, Laboratoire de Bactériologie, CNR des Campylobacters et des Hélicobacters, Place Amélie Raba Léon, 33076 Bordeaux Cedex, France

**Keywords:** *H. pylori*, PCR, Clarithromycin resistance, ELITe InGenius, Automation

## Abstract

PCR detection of *Helicobacter pylori* infection in gastric biopsies allows the detection of this bacterium and the mutations associated with macrolide resistance. The aim of this study was to evaluate the performance of RIDA®GENE *H. pylori* PCR (r-Biopharm) on the ELITe InGenius System (Elitech). Two hundred gastric biopsies were obtained. These biopsies were ground in nutrient broth. Two hundred microliters of this suspension was treated with proteinase K, and then, 200 µL was transferred to an ELITe InGenius sample tube and tested using RIDA®GENE *H. pylori* PCR reagents. In-house *H. pylori* PCR was used as a reference. The sensitivity of RIDA®GENE *H. pylori* PCR with ELITe InGenius was 100%, the specificity was 98% (95% confidence interval (CI), 95.3–100%), the PPV was 98% (95% CI, 95.3–100%), and the NPV was 100% for the detection of *H. pylori*. All of these parameters were 100% for the categorization of macrolide resistance. The adaptation of RIDA®GENE *H. pylori* PCR reagents on the ELITe InGenius System was successful. This PCR is easy to use on this system.

**Supplementary Information:**

The online version contains supplementary material available at 10.1007/s10096-023-04563-3.

## Introduction

Of all the techniques available for the diagnosis of *Helicobacter pylori* infection in gastric biopsies, PCR is now widely used in microbiology laboratories. It is more sensitive than culture [1] and provides information on the presence of infection and macrolide resistance mutations within hours. Many commercial kits have been developed, and their performances have been evaluated. Our laboratory has participated in the evaluation of some of them [2–4].

We previously demonstrated that RIDA®GENE *H. pylori* PCR (r-Biopharm, Courtaboeuf, France) [2] could be adapted to the BD MAX™ device commercialized by Becton Dickinson (Le Pont de Claix, France) [5, 6]. This kind of system has the advantage of full automation of DNA extraction, PCR amplification, and analysis of the results. The aim of our study was to evaluate RIDA®GENE *H. pylori* PCR on an equivalent system, the ELITe InGenius from the Elitech Group (Puteaux, France). This evaluation was performed retrospectively using 200 frozen gastric biopsies with known *H. pylori* status and macrolide sensitivity.

## Material and methods

### Study design

The procedure for the ELITe InGenius System was optimized for the isolation of DNA from 200 µL samples. ELITe InGenius contains a combination of lytic and extraction reagents designed to perform cell lysis and DNA extraction. Following cell lysis, the released DNA is captured by magnetic affinity beads. The beads with the bound DNA were washed, and then, the DNA was eluted using 100 μL of elution buffer. The eluted DNA may be used for applications on the ELITe InGenius. The PCR amplification parameters used on the ELITe InGenius were denaturation at 95 °C for 60 s (1 cycle) followed by 95 °C for 15 s and 60 °C for 30 s of amplification and detection (45 cycles). Channels 1 (Ct threshold 50), 2 (Ct threshold 50), and 5 (Ct threshold 200) were used for *H. pylori,* internal control (IC), and clarithromycin resistance detection, respectively.

Two hundred gastric biopsies obtained from the National Reference Center for Campylobacters and Helicobacters (NRCCH) (www.cnrch.fr) received at the NRCCH during April and July 2021 were included (Suppl Table 1). They were acquired from 105 women and 95 men (sex ratio 0.47) with an average age of 49 years ± 16.9. On receipt, according to a routine protocol, these biopsies were previously ground in 1 mL of nutrient broth and then stored at −80 °C. Part of this suspension was treated with 20 μL of proteinase K (pK) (Roche Diagnostic, Meylan, France) + 180 μL of ATL Qiagen buffer at 56 °C for 3 h, and 200 µL was used for the test on the ELITe InGenius. The same digestion protocol was used on ground biopsies before DNA extraction on a MagNA Pure 96 system (Roche Diagnostics) and PCR on Eurogentec strips (Liège, Belgium) using an LC480 (Roche Diagnostics, Meylan, France) as previously described [7]. The culture was performed in parallel according to internal laboratory procedures [8].

### Limits of detection

The reference *H. pylori* strain CCUG17874 (susceptible to clarithromycin) and a clinical strain from a routine procedure (isolated from the gastric biopsy of a 63-year-old man) resistant to clarithromycin with an A2142 or A2143G mutation were grown on homemade Pylori agar [8] and then used to evaluate the limit of detection of RIDA®GENE PCR on the ELITe InGenius. Both strains were suspended at 1.5 McF (approximately 1.4×10^8^ CFU/mL) in nutrient Brucella broth, and then, serial dilutions from 10^−4^ to 10^−7^ were generated. Two hundred microliters of each dilution was used for extraction and PCR analysis on the ELITe InGenius.

### Reference used

*H. pylori* PCR from NRCCH was used as a reference [7]. In the event of a discrepancy, each biopsy was tested a second time on the ELITe InGenius, and PCR on the DNA extracted by the NRCCH with the MagNA Pure 96 extractor on a CFX96 system (Bio-Rad, Les Ulis, France) using RIDA®GENE *H. pylori* reagents was performed as previously described [2]. In the event of an unresolved discrepancy, clinical information was considered if available.

## Results

Of the 200 biopsies, 100 were expected to be negative for *H. pylori*. In all, 98 biopsies were negative on the ELITe InGenius using RIDA®GENE *H. pylori* reagents (Table 1) (Suppl Table 1). Two biopsies were positive for *H. pylori* (biopsies 159 and 189, with Ct values of 35 and 38.4, respectively) (Suppl Table 1). The amplification curves of these two samples were similar to those obtained on the other *H. pylori* positive samples. These two cases were detected as positive again on a second passage on the ELITe InGenius. The 2 DNA samples extracted at the NRCCH for these 2 cases tested negative using RIDA®GENE *H. pylori* reagents on a CFX96 system (data not shown). No history or suspicion of *H. pylori* infection was available for these other discordant cases, and thus, they were considered false-positives (Table 1) (Table 2).

**Table 1:** Summary of the results obtained from the 200 gastric biopsies tested

In-house PCR	PCR ELITe InGenius	No.
Neg	Neg	98
Neg	Pos-WT	2
Pos-WT	Pos-WT	65
Pos-A2142-3G	Pos-mutated	30
Pos-WT+A2142-3G	Pos-mutated	5
Total		200

*Neg*, negative; *Pos*, positive; *Ct*, threshold; *WT*, wild-type; *A2142-3G*, A2142G or A2143G mutation

**Table 2:** Performance of the RIDA®GENE *H. pylori* PCR on the ELITe InGenius

Target	In-house result	ELITe InGenius PCR result		Total
*H. pylori*		POS	NEG	
	POS	100	0	100
	NEG	2	98	100
23S rDNA genotype		WT	Mutated	
	WT	65	0	65
	Mutated	0	35	35

In-house *H. pylori* PCR was used as a reference [7]. In the event of a discrepancy in *H. pylori* detection, the clinical history of *H. pylori* infection was considered. In the event of a discrepancy in the sensitivity to macrolides, the antibiogram result was considered

*WT*, wild-type; *mutated*, presence of 23S rDNA mutations; *POS*, *H. pylori* positive; *NEG*, *H. pylori* negative

The 100 biopsies expected to be positive for *H. pylori* according to the NRCCH results were also positive on the ELITe InGenius using RIDA®GENE *H. pylori* reagents. The mean Ct value for *H. pylori* detection for these 100 biopsies was 23.3 Ct ± 2.5. The performance of RIDA®GENE *H. pylori* PCR on an ELITe InGenius for *H. pylori* detection was therefore determined to be as follows: sensitivity 100%, specificity 98% (95% confidence interval (CI), 95.3.21–100%), negative predictive value (NPV) 100%, and positive predictive value (PPV) 98% (95% CI, 95.3–100%).

The limit of detection of *H. pylori* (see “Material and methods”) on the ELITe InGenius was approximately 65 CFU (data not shown).

Regarding the detection on the ELITe InGenius of the 23S rDNA genotype associated with sensitivity to macrolides, a population sensitive to macrolides (WT genotype) was expected for 65 biopsies: these samples were perfectly detected and categorized on the ELITe InGenius. For 30 biopsies, *H. pylori* with an A2142G or A2143G mutation was expected: they were perfectly detected and categorized on the ELITe InGenius. For 5 biopsies, a mixed WT + A2142G or A2143G-mutated population was expected (Table 1) (Table 2) (Suppl Table 1), and they were perfectly categorized.

The mean Ct value for detecting clarithromycin resistance was 26.7 ± 2.4. The detection performance of the 23S rDNA genotype on the ELITe InGenius was therefore perfect, with 100% sensitivity, specificity, NPV, and PPV.

## Discussion

The objective of our study was to evaluate RIDA®GENE *H. pylori* PCR on an ELITe InGenius using tissue from gastric biopsies. The performance validation was conducted by a retrospective study of 200 gastric biopsies. Our results showed that this adaptation is possible, and the results obtained were excellent.

The advantage of automated RIDA®GENE *H. pylori* PCR on an ELITe InGenius is to provide the possibility for clinical laboratories equipped with this system to detect not only *H. pylori* but also the mutations associated with macrolide resistance. Interpretation of the results, which is possible on the ELITe InGenius, was easy. We did not have to adjust the detection of the IC, which is an essential parameter used to interpret the results, particularly for negative samples. IC amplification can be inhibited if *H. pylori* is detected, but this does not interfere with interpretation. If only macrolide resistance is detected, i.e., in the absence of *H. pylori* detection*,* the test, as indicated by r-Biopharm, should be interpreted as negative. For the 100 biopsies expected to be negative according to the in-house PCR, this situation occurred only once (biopsy 142) (Suppl Table 1).

The discrepancies in this study, compared with our real-time PCR method used as a reference, were observed only in 2 samples. Two false-positive results were indeed detected for which histology reports were not available. RIDA®GENE *H. pylori* PCR on an ELITe InGenius correctly detects the mutations associated with macrolide resistance (point mutations at two nucleotide positions, 2142 (A2142G and A2142C) and 2143 (A2143G) [1]), even for biopsies containing a mixture of susceptible and resistant populations. However, it would be interesting to verify these results in a larger number of biopsies of this type. The A2142G or A2143G mutations are the most frequent in France, and in Europe, the A2142C mutation remains anecdotal [9, 7]. Finally, A2142T and A2143C mutations are rarely found in France (<0.1% of all macrolide-resistant strains in France (2017–2021 personal data)) [10]. Samples positive for these mutations (one sample each) were tested on the ELITe InGenius; as expected, *H. pylori-*only was detected (data not shown).

In conclusion, the performance of RIDA®GENE *H. pylori* PCR on the ELITe InGenius was excellent. The performance was similar to that published previously by our team for these same reagents but with a different DNA extraction technique and an independent real-time PCR device, the CFX96 [2] or the BD Max system [6]. This adaptation in such apparatuses allows the automation of DNA extraction, PCR amplification, and automatic interpretation of results in a single machine. The advantage of the ELITe InGenius System is to be able to recover DNA extracted by the system for controls or further analysis if needed, whereas this is not possible on the BD MAX™ where the DNA at the end of the run is mixed with the reaction mix. The capacity of the BD MAX™ is 24 samples against only 12 for the ELITe InGenius. The choice of the machine will depend on the volume of activity of each laboratory wishing to set up this PCR. The reading and interpretation of the amplification curves and the results obtained is easier on BD MAX™ than on ELITe InGenius. On the other hand, the launching of an analysis run on ELITe InGenius is guided step by step by the machine whereas it is less intuitive on BD MAX™. The number of reagents needed on BD MAX™ is however smaller than on ELITe InGenius. In the end, each system has its advantages and disadvantages which can be assessed by future users according to their working environment. Their use is very simple, and the obtained performances are excellent.

## Supplementary Information

Below is the link to the electronic supplementary material.

Supplementary file1 (DOCX 66 KB)

## Data Availability

The datasets generated during and/or analyzed during the current study are available from the corresponding author on reasonable request.
