# Supplementary material for: RIDA®GENE Helicobacter pylori PCR on the ELITe InGenius System
Source: Eur J Clin Microbiol Infect Dis. 2023 Mar 16;42(5):593–6. doi: 10.1007/s10096-023-04563-3 (PMC10105649; doi:10.1007/s10096-023-04563-3)
Supplement: Supplementary file 1 — Supplementary file1 (DOCX 66 KB) [file 10096_2023_4563_MOESM1_ESM.docx]

**Suppl Table 1.** Results obtained from the 200 gastric biopsies included in the prospective study.

| **No.** | **Sex** | **Age** | **In-house PCR** | **Ct** | **Genotype** | **ELITe InGenius PCR** | **Ct** | **Cla-R** | **Ct** | **IC** |
| --- | --- | --- | --- | --- | --- | --- | --- | --- | --- | --- |
| 1 | **2** | **59** | + | 21.2 | G | + | 17.2 | + | 21.7 | - |
| 2 | **2** | **53** | + | 24.1 | G | + | 21.6 | + | 25.7 | - |
| 3 | **1** | **11** | + | 24.9 | G | + | 20.3 | + | 24.7 | - |
| 4 | **2** | **44** | + | 26.3 | G | + | 23.2 | + | 27.3 | 34.1 |
| 5 | **1** | **46** | + | 28 | G | + | 23.3 | + | 27.7 | 31.9 |
| 6 | **2** | **54** | + | 25 | G | + | 26.3 | + | 30.9 | 28 |
| 7 | **2** | **6** | + | 26.2 | G | + | 23.3 | + | 27.5 | 29.5 |
| 8 | **2** | **52** | + | 23.5 | G | + | 18.7 | + | 22.6 | - |
| 9 | **2** | **56** | + | 29 | G | + | 20 | + | 24.1 | - |
| 10 | **1** | **60** | + | 26.6 | G | + | 27.9 | + | 32.1 | 29 |
| 11 | **2** | **51** | + | 28.9 | G | + | 23.8 | + | 27.6 | - |
| 12 | **1** | **48** | + | 29.8 | WT+G | + | 21.9 | + | 25.7 | - |
| 13 | **2** | **60** | + | 24.5 | G | + | 20.3 | + | 23.9 | - |
| 14 | **2** | **30** | + | 24.9 | G | + | 20.1 | + | 24.1 | - |
| 15 | **2** | **77** | + | 25 | G | + | 21.1 | + | 24.9 | - |
| 16 | **1** | **17** | + | 32.1 | G | + | 23 | + | 27.1 | - |
| 17 | **2** | **44** | + | 25.7 | WT+G | + | 20.3 | + | 26.1 | - |
| 18 | **2** | **50** | + | 27.7 | G | + | 24.4 | + | 28.6 | 28.5 |
| 19 | **2** | **63** | + | 22.8 | WT | + | 22.3 | - | - | 31.8 |
| 20 | **1** | **81** | + | 21 | G | + | 19.1 | + | 23.4 | - |
| 21 | **2** | **57** | + | 32.6 | G | + | 26.3 | + | 30.2 | 29.1 |
| 22 | **1** | **34** | + | 24.9 | WT | + | 20.1 | - | - | - |
| 23 | **1** | **32** | + | 24.3 | WT | + | 24.8 | - | - | 30.7 |
| 24 | **2** | **64** | + | 28 | G | + | 27.8 | + | 32.1 | 28.5 |
| 25 | **2** | **46** | + | 21.6 | WT | + | 22.2 | - | - | 30.5 |
| 26 | **2** | **62** | + | 24.3 | WT | + | 27.3 | - | - | 27.5 |
| 27 | **1** | **76** | + | 23.7 | WT | + | 31.7 | - | - | 28.1 |
| 28 | **2** | **34** | + | 21.8 | WT | + | 26.1 | - | - | 27.9 |
| 29 | **2** | **69** | + | 24 | WT | + | 29.5 | - | - | 27.6 |
| 30 | **2** | **28** | + | 20 | WT | + | 20.5 | - | - | - |
| 31 | **2** | **57** | + | 23.6 | WT | + | 24.7 | - | - | 28.8 |
| 32 | **2** | **41** | + | 27.8 | G | + | 23 | + | 26.8 | 32.8 |
| 33 | **1** | **52** | + | 25 | G | + | 27.1 | + | 30.9 | 28.5 |
| 34 | **1** | **52** | + | 25.3 | WT+G | + | 25.4 | + | 30.3 | 28.6 |
| 35 | **1** | **53** | + | 19.8 | WT | + | 21.8 | - | - | - |
| 36 | **1** | **63** | + | 24.6 | WT | + | 22.1 | - | - | 33.3 |
| 37 | **2** | **39** | + | 20.7 | WT | + | 22.7 | - | - | - |
| 38 | **2** | **69** | + | 20.3 | WT | + | 22.2 | - | - | - |
| 39 | **1** | **54** | + | 24 | WT | + | 22.6 | - | - | 29.7 |
| 40 | **2** | **32** | + | 20.4 | WT | + | 21.5 | - | - | - |
| 41 | **1** | **37** | + | 18.4 | WT | + | 20.1 | - | - | - |
| 42 | **2** | **84** | + | 31.7 | WT | + | 26.9 | - | - | 28.6 |
| 43 | **1** | **44** | + | 21.9 | WT | + | 21 | - | - | 32.3 |
| 44 | **1** | **74** | + | 23.3 | WT | + | 21.4 | - | - | 37.1 |
| 45 | **2** | **49** | + | 26.2 | WT | + | 21.7 | - | - | - |
| 46 | **2** | **78** | + | 25.4 | WT | + | 21.4 | - | - | 37.1 |
| 47 | **1** | **21** | + | 24 | WT | + | 20.8 | - | - | - |
| 48 | **2** | **34** | + | 23 | WT | + | 20 | - | - | - |
| 49 | **2** | **10** | + | 23.5 | WT | + | 18.3 | - | - | - |
| 50 | **2** | **48** | + | 22.7 | WT | + | 23.6 | - | - | 32.9 |
| 51 | **1** | **69** | + | 27 | WT | + | 21.8 | - | - | - |
| 52 | **1** | **33** | + | 22.6 | WT | + | 20.2 | - | - | - |
| 53 | **1** | **25** | + | 23.9 | WT | + | 23.4 | - | - | 31.4 |
| 54 | **1** | **81** | + | 27.9 | G | + | 25 | + | 29 | 29.2 |
| 55 | **1** | **30** | + | 24.3 | WT | + | 23.4 | - | - | 32.6 |
| 56 | **2** | **46** | + | 35.2 | WT | + | 38 | - | - | 29.1 |
| 57 | **2** | **66** | + | 21.5 | WT | + | 21.9 | - | - | - |
| 58 | **1** | **16** | + | 29.5 | WT | + | 25.3 | - | - | 29.3 |
| 59 | **1** | **8** | + | 23 | G | + | 19.7 | + | 24.2 | - |
| 60 | **2** | **56** | + | 26 | WT | + | 24.2 | - | - | 32.9 |
| 61 | **2** | **30** | + | 26.8 | G | + | 24.6 | + | 28.8 | 30.5 |
| 62 | **2** | **37** | + | 24 | WT | + | 21.3 | - | - | - |
| 63 | **2** | **69** | + | 20.9 | WT | + | 19.2 | - | - | - |
| 64 | **1** | **69** | + | 29.7 | WT | + | 23.3 | - | - | 33.4 |
| 65 | **1** | **70** | + | 28.7 | WT | + | 24.1 | - | - | 31.2 |
| 66 | **1** | **46** | + | 28.5 | WT | + | 24.4 | - | - | 28.2 |
| 67 | **2** | **80** | + | 23.6 | WT | + | 22.7 | - | - | - |
| 68 | **2** | **38** | + | 24.6 | WT | + | 19 | - | - | - |
| 69 | **1** | **11** | + | 28.0 | WT | + | 25.6 | - | - | - |
| 70 | **2** | **44** | + | 25.9 | WT | + | 24.3 | - | - | 29.8 |
| 71 | **2** | **28** | + | 22.1 | WT | + | 19.2 | - | - | - |
| 72 | **1** | **63** | + | 23.5 | WT | + | 21.4 | - | - | - |
| 73 | **2** | **47** | + | 23 | G | + | 21.5 | + | 25.5 | - |
| 74 | **1** | **22** | + | 20.5 | WT+G | + | 18.5 | + | 25.7 | - |
| 75 | **1** | **74** | + | 20.8 | WT | + | 26.7 | - | - | 27.9 |
| 76 | **1** | **71** | + | 21 | WT | + | 20.7 | - | - | - |
| 77 | **2** | **43** | + | 33.6 | WT | + | 35.3 | - | - | 28.1 |
| 78 | **1** | **66** | + | 21.3 | WT | + | 22.9 | - | - | - |
| 79 | **1** | **66** | + | 18.7 | WT | + | 18.1 | - | - | - |
| 80 | **2** | **61** | + | 32 | WT | + | 31.5 | - | - | 27.8 |
| 81 | **1** | **75** | + | 24 | WT | + | 20.6 | - | - | - |
| 82 | **2** | **54** | + | 20.2 | G | + | 18.4 | + | 21.9 | - |
| 83 | **2** | **79** | + | 23 | G | + | 27 | + | 31.1 | 28.5 |
| 84 | **1** | **35** | + | 30.2 | WT | + | 23.4 | - | - | - |
| 85 | **1** | **37** | + | 25.3 | WT | + | 23.4 | - | - | 30.5 |
| 86 | **1** | **69** | + | 22.3 | WT | + | 17.8 | - | - | - |
| 87 | **1** | **36** | + | 24.2 | WT | + | 24.3 | - | - | 30.5 |
| 88 | **1** | **32** | + | 24 | WT | + | 21.5 | - | - | - |
| 89 | **1** | **34** | + | 24.3 | WT | + | 27 | - | - | 28.1 |
| 90 | **2** | **68** | + | 20 | G | + | 19.6 | + | 23.7 | - |
| 91 | **1** | **73** | + | 34 | G | + | 21.9 | + | 26.7 | - |
| 92 | **2** | **40** | + | 21.5 | WT+G | + | 24 | + | 28.3 | 32.5 |
| 93 | **1** | **48** | + | 27.1 | WT | + | 24.7 | - | - | 28.5 |
| 94 | **1** | **47** | + | 24.4 | WT | + | 23.1 | - | - | - |
| 95 | **1** | **25** | + | 26.7 | WT | + | 23.7 | - | - | - |
| 96 | **2** | **13** | + | 24.2 | WT | + | 22.8 | - | - | - |
| 97 | **2** | **17** | + | 28.2 | WT | + | 25.5 | - | - | 30 |
| 98 | **1** | **35** | + | 27.1 | WT | + | 22.8 | - | - | - |
| 99 | **1** | **74** | + | 22.8 | WT | + | 20 | - | - | - |
| 100 | **1** | **68** | + | 21.8 | G | + | 21.2 | + | 25.3 | - |
| 101 | **1** | **29** | - | / | / | - | - | - | - | 28.1 |
| 102 | **1** | **42** | - | / | / | - | - | - | - | 28.5 |
| 103 | **2** | **52** | - | / | / | - | - | - | - | 27.7 |
| 104 | **1** | **9** | - | / | / | - | - | - | - | 28.4 |
| 105 | **1** | **30** | - | / | / | - | - | - | - | 28.6 |
| 106 | **1** | **36** | - | / | / | - | - | - | - | 28.3 |
| 107 | **2** | **40** | - | / | / | - | - | - | - | 27.9 |
| 108 | **1** | **78** | - | / | / | - | - | - | - | 27.8 |
| 109 | **1** | **30** | - | / | / | - | - | - | - | 28.3 |
| 110 | **1** | **49** | - | / | / | - | - | - | - | 28.2 |
| 111 | **2** | **26** | - | / | / | - | - | - | - | 28.2 |
| 112 | **2** | **65** | - | / | / | - | - | - | - | 28.2 |
| 113 | **2** | **40** | - | / | / | - | - | - | - | 28.3 |
| 114 | **2** | **40** | - | / | / | - | - | - | - | 28.4 |
| 115 | **2** | **46** | - | / | / | - | - | - | - | 28.5 |
| 116 | **2** | **70** | - | / | / | - | - | - | - | 28.3 |
| 117 | **1** | **34** | - | / | / | - | - | - | - | 28 |
| 118 | **1** | **22** | - | / | / | - | - | - | - | 28 |
| 119 | **2** | **63** | - | / | / | - | - | - | - | 28.5 |
| 120 | **1** | **28** | - | / | / | - | - | - | - | 29.2 |
| 121 | **2** | **34** | - | / | / | - | - | - | - | 29 |
| 122 | **2** | **36** | - | / | / | - | - | - | - | 28.8 |
| 123 | **1** | **43** | - | / | / | - | - | - | - | 29.2 |
| 124 | **2** | **15** | - | / | / | - | - | - | - | 28.9 |
| 125 | **1** | **55** | - | / | / | - | - | - | - | 29.6 |
| 126 | **1** | **80** | - | / | / | - | - | - | - | 29 |
| 127 | **1** | **16** | - | / | / | - | - | - | - | 29.8 |
| 128 | **2** | **35** | - | / | / | - | - | - | - | 29.2 |
| 129 | **2** | **75** | - | / | / | - | - | - | - | 28.7 |
| 130 | **1** | **0** |  | / | / | - | - | - | - | 28.6 |
| 131 | **1** | **65** |  | / | / | - | - | - | - | 29.1 |
| 132 | **2** | **70** | - | / | / | - | - | - | - | 28.6 |
| 133 | **2** | **52** | - | / | / | - | - | - | - | 28.6 |
| 134 | **1** | **33** | - | / | / | - | - | - | - | 28.8 |
| 135 | **2** | **28** | - | / | / | - | - | - | - | 28.7 |
| 136 | **2** | **24** | - | / | / | - | - | - | - | 28.7 |
| 137 | **1** | **55** | - | / | / | - | - | - | - | 28.4 |
| 138 | **2** | **29** | - | / | / | - | - | - | - | 28.3 |
| 139 | **1** | **77** | - | / | / | - | - | - | - | 28.9 |
| 140 | **2** | **40** | - | / | / | - | - | - | - | 29.5 |
| 141 | **1** | **71** | - | / | / | - | - | - | - | 30 |
| 142 | **2** | **54** | - | / | / | - | - | ! | 41.9 | 29.5 |
| 143 | **2** | **22** | - | / | / | - | - | - | - | 28.5 |
| 144 | **2** | **32** | - | / | / | - | - | - | - | 29 |
| 145 | **2** | **17** | - | / | / | - | - | - | - | 29.2 |
| 146 | **2** | **40** | - | / | / | - | - | - | - | 28.9 |
| 147 | **2** | **42** | - | / | / | - | - | - | - | 29.1 |
| 148 | **2** | **27** | - | / | / | - | - | - | - | 29.2 |
| 149 | **1** | **71** | - | / | / | - | - | - | - | 28.9 |
| 150 | **2** | **52** | - | / | / | - | - | - | - | 28.6 |
| 151 | **2** | **42** | - | / | / | - | - | - | - | 28.9 |
| 152 | **2** | **58** | - | / | / | - | - | - | - | 29.3 |
| 153 | **2** | **42** | - | / | / | - | - | - | - | 29.1 |
| 154 | **1** | **58** | - | / | / | - | - | - | - | 29.2 |
| 155 | **1** | **71** | - | / | / | - | - | - | - | 29.8 |
| 156 | **1** | **52** | - | / | / | - | - | - | - | 29 |
| 157 | **2** | **13** | - | / | / | - | - | - | - | 27.9 |
| 158 | **2** | **45** | - | / | / | - | - | - | - | 28.3 |
| 159 | **1** | **50** | - | / | / | + | 35 | - | - | 28.1 |
| 160 | **2** | **67** | - | / | / | - | - | - | - | 28 |
| 161 | **1** | **35** | - | / | / | - | - | - | - | 28.6 |
| 162 | **2** | **67** | - | / | / | - | - | - | - | 28.2 |
| 163 | **2** | **37** | - | / | / | - | - | - | - | 28.4 |
| 164 | **2** | **57** | - | / | / | - | - | - | - | 28.8 |
| 165 | **1** | **33** | - | / | / | - | - | - | - | 28 |
| 166 | **2** | **54** | - | / | / | - | - | - | - | 28.1 |
| 167 | **2** | **73** | - | / | / | - | - | - | - | 28.3 |
| 168 | **1** | **77** | - | / | / | - | - | - | - | 28.3 |
| 169 | **2** | **34** | - | / | / | - | - | - | - | 28.1 |
| 170 | **1** | **79** | - | / | / | - | - | - | - | 28.1 |
| 171 | **1** | **78** | - | / | / | - | - | - | - | 28.4 |
| 172 | **1** | **27** | - | / | / | - | - | - | - | 28.3 |
| 173 | **2** | **85** | - | / | / | - | - | - | - | 28.3 |
| 174 | **2** | **36** | - | / | / | - | - | - | - | 28.4 |
| 175 | **1** | **74** | - | / | / | - | - | - | - | 28.4 |
| 176 | **1** | **68** | - | / | / | - | - | - | - | 28.1 |
| 177 | **1** | **70** | - | / | / | - | - | - | - | 28.9 |
| 178 | **1** | **62** | - | / | / | - | - | - | - | 28.6 |
| 179 | **1** | **41** | - | / | / | - | - | - | - | 28.4 |
| 180 | **1** | **24** | - | / | / | - | - | - | - | 28.2 |
| 181 | **2** | **25** | - | / | / | - | - | - | - | 28.5 |
| 182 | **2** | **18** | - | / | / | - | - | - | - | 28.4 |
| 183 | **2** | **37** | - | / | / | - | - | - | - | 28.5 |
| 184 | **2** | **73** | - | / | / | - | - | - | - | 28.4 |
| 185 | **2** | **42** | - | / | / | - | - | - | - | 28.3 |
| 186 | **2** | **36** | - | / | / | - | - | - | - | 29.2 |
| 187 | **2** | **73** | - | / | / | - | - | - | - | 28.3 |
| 188 | **2** | **24** | - | / | / | - | - | - | - | 28.7 |
| 189 | **1** | **51** | - | / | / | - | - | - | - | 28.8 |
| 190 | **1** | **57** | - | / | / | - | - | - | - | 29.4 |
| 191 | **2** | **42** | - | / | / | - | - | - | - | 28.3 |
| 192 | **1** | **83** | - | / | / | - | - | - | - | 29.2 |
| 193 | **1** | **66** | - | / | / | - | - | - | - | 29 |
| 194 | **2** | **71** | - | / | / | - | - | - | - | 29.3 |
| 195 | **1** | **61** | - | / | / | - | - | - | - | 29.3 |
| 196 | **2** | **52** | - | / | / | - | - | - | - | 29 |
| 197 | **1** | **68** | - | / | / | - | - | - | - | 28.5 |
| 198 | **1** | **83** | - | / | / | - | - | - | - | 28 |
| 199 | **1** | **80** | - | / | / | + | 38.4 | - | - | 28.5 |
| 200 | **2** | **76** | - | / | / | - | - | - | - | 28.9 |

Ct: threshold; Cla-R: clarithromycin resistance detected by BD MAX^TM^; IC: internal control. Gray: discrepant results; !: detection of macrolide resistance in the absence of *H. pylori* detection*.*

WT: wild-type; G: A2142 or A2143G mutation.
